# Nursing care for hospitalized older adults - fall accidents versus safe mobility: a scoping review

**DOI:** 10.1590/0034-7167-2023-0180

**Published:** 2024-07-19

**Authors:** Esther Mourão Nicoli, Frances Valéria Costa e Silva, Luciana Guimarães Assad, Camila Castanho Cardinelli, Raquel Azevedo Alves, Samara Gonçalves de Oliveira

**Affiliations:** I Universidade do Estado do Rio de Janeiro. Rio de Janeiro, Rio de Janeiro, Brazil

**Keywords:** Accidental Falls, Nursing Care, Mobility Limitation, Aged, Hospitals, Accidentes por Caídas, Atención de Enfermería, Limitación de la Movilidad, Anciano, Atención Hospitalaria

## Abstract

**Objectives:**

to map the constituent elements of the safe mobility concept present in hospital care for older adults.

**Methods:**

a scoping review of 35 articles searched in databases and gray literature - BDENF/VHL, Scopus, CINAHL/EBSCO, Embase, Web of Science, PEDro, MEDLINE/PubMed and CAPES Theses and Dissertations Catalog. No time or language cut-off was established.

**Results:**

none of the studies presented a clear safe mobility concept, however its constituent elements involve factors related to patient (behavioral factors, conditions, diseases, signs and symptoms, nutritional status, age, balance, strength, gait quality, sleep), the institution (environment, treatment devices, guidelines, medications and polypharmacy, material and human resources and clothing/shoes) and the nature of the interventions (related to the patient, institution and family).

**Final Considerations:**

the constituent elements of safe mobility express hospital units’ capacity to guarantee care and protection from fall accidents for hospitalized older adults.

## RESUMEN

**Objetivos:**

mapear los elementos constitutivos del concepto de movilidad segura presente en la atención hospitalaria a las personas mayores.

**Métodos:**

revisión de alcance de 35 artículos buscados en bases de datos y literatura gris - BDENF/VHL, Scopus, CINAHL/EBSCO, Embase, *Web of Science*, PEDro, MEDLINE/PubMed y Catálogo CAPES de Tesis y Disertaciones. No se estableció ningún límite de tiempo ni de idioma.

**Resultados:**

ninguno de los estudios presentó un concepto claro de movilidad segura, sin embargo sus elementos constitutivos involucran factores relacionados con el paciente (factores de comportamiento, condiciones, enfermedades, signos y síntomas, estado nutricional, edad, equilibrio, fuerza, calidad de la marcha, sueño), la institución (ambiente, dispositivos de tratamiento, pautas, medicamentos y polifarmacia, recursos materiales y humanos y vestimenta/calzado) y la naturaleza de las intervenciones (relacionadas con el paciente, la institución y la familia).

**Consideraciones Finales:**

los elementos constitutivos de la movilidad segura expresan la capacidad de las unidades hospitalarias para garantizar la atención y protección contra accidentes por caídas a los ancianos hospitalizados.

## INTRODUCTION

The occurrence of falls in older adults, despite continuous nursing efforts to reduce them, still represents one of the most important incidents in hospital environments, considering its frequency, economic impacts and consequences^(1)^.

This situation arises from the fall prevention programs and protocols implemented, which tend to simplify the event and overvalue prevention itself, neglecting fall risk assessment in a care plan^(2)^. Consequently, there is a greater emphasis on summing up and recording the scores obtained through the application of fall predictive scales, while less time is dedicated to proposing interventions that address modifiable risk factors^(2)^.

During the last four decades, nursing practices related to fall prevention have not undergone significant changes^(3)^. Usually, the measures adopted focus on reducing patient mobility or improving environmental safety, without considering that intrinsic factors are, in fact, the main precursors to falls^(2)^.

However, interventions that limit mobility, such as signaling bracelets, recommendations for bed rest or reduced time spent using the toilet, despite offering some protection against falls, can lead to functional decline and the development of geriatric syndromes, such as instability postural, immobility and iatrogenesis^(4)^. Furthermore, such restrictive interventions increase the length of hospital stay, re-hospitalization risk and the likelihood of complications that, in turn, exacerbate future fall risk^(4)^.

During the hospitalization period, patients remain sitting or lying down on average 87% to 100% of the time^(5)^. Therefore, in older adults, even if healthy, ten days of bed rest are equivalent to a reduction of approximately 12% in aerobic capacity and 16% in the strength of the knee extensor muscles, which impacts a significant decline in functional capacity^(2)^.

In this context, the “safe mobility” concept expresses a transition in perspective, assessment and recognition of fall risk factors^(2)^. There is, therefore, an emphasis on intrinsic factors, without, in turn, disregarding protocols, predictive scales and extrinsic factors, which are also important, integrating evidence into care plan with an interand multi-professional approach^(2)^.

Safe mobility is centered on patients, valuing their individuality, preferences, needs and values^(6)^. Therefore, older adults are properly informed and consulted so that shared decisions can then be made that consider their wishes, technical issues and scientific evidence^(6)^. When older adults actively participate in planning their care, they can become aware of the risks and their own condition, which, in turn, motivates them to adopt safe mobility behaviors^(6)^.

This approach therefore implies greater critical thinking^(2)^. It is essential to carefully assess bed restriction guidelines, in addition to considering that preserving mobility may involve some risk of falling^(2)^. However, a realistic mobility plan may be more beneficial to older adults’ well-being than the effort to avoid falls at any cost^(2)^.

Nurses, as care managers and influential agents in this context, play a fundamental role in preventing falls^(7)^. They are responsible for introducing and disseminating interventions that consider the individual needs of each older adult, aiming for their well-being, safety and autonomy^(7)^. From this view, clarifying the understanding of the constituent elements of the “safe mobility” concept expands the possibility of constructing nursing interventions aligned with this objective, which is a knowledge gap.

In January 2022, a preliminary search was conducted in the PubMed, CINAHL and JBI databases, which revealed a scarcity of studies on the topic. This motivated a proposal to prepare a scoping review in order to map documents addressing the term “safe mobility” in the literature^(8)^. Such a study strengthens the movement of scientific production in the highlighted thematic universe, since a scoping review makes it possible to identify gaps in the literature, clarify concepts and summarize findings, in addition to systematizing and disseminating findings that can contribute to practices, policies and research^(8)^.

## OBJECTIVES

To map the constituent elements of safe mobility present in hospital care for older adults.

## METHODS

### Study design

This is a scoping review study guided by JBI guidelines, an international research organization that guides systematic reviews^(9)^. Initially, the review question was established, structured by the acronym PCC - P (population/participant), C (concept) and C (context)^(10)^: what are the constituent elements of the safe mobility concept in hospitalized older adults at risk of falling present in national and international studies?

The review protocol was registered in the Open Science Framework (OSF), under DOI 10.17605/OSF.IO/EDHF6, and subsequently published in the Online Brazilian Journal of Nursing (OBJN)^(11)^.

Valuing the writing quality and smoothness of this study, the Preferred Reporting Items for Systematic reviews and Meta-Analyses extension for Scoping Reviews (PRISMA-ScR) checklist guidelines were followed^(12)^.

### Eligibility criteria

The eligibility criteria are linked to the PCC acronym structure. For population/participant, older adults - individuals aged 60 or older, according to the Older Adult Statute classification (Law 14,423 of 2022)^(13)^ - of both sexes were included. For concept, studies that define, report or provide information on safe mobility - relevant approaches that help or encourage older adults to move safely daily, aiming to preserve functional capacity - and the factors associated with promoting this were included. Studies dealing with urban mobility were excluded. For context, studies that involve the care of hospitalized older adults, in multiple circumstances (clinical, surgical, among others), covering public or private hospitals, small, medium or large, teaching, general, specialized, urban or rural, were included.

The review considered primary research studies, systematic reviews, meta-syntheses and case reports, with a quantitative or qualitative design. Furthermore, reports, institutional texts with relevance in geriatrics/gerontology, books and guidelines published in indexed sources consulted or in gray literature were included. Articles published only as abstracts, letters to the editor and comments were excluded. No time and language cut-off were established.

### Data collect

For data collection, a three-step search strategy was developed. The initial stage, carried out in April 2022, consisted of identifying the search terms, and, to this end, controlled words in health in DeCS (Health Sciences Descriptors), MeSH (Medical Subjective Headings) and Emtree were consulted (Embase Subject Headings). The following terms were included:

P (population/participant) - middle aged (*pessoa de meia-idade*)/aged (*idoso*)/old people (*pessoa idosa*)/old person (*pessoa idos*a)/elderly (*idoso*)/Elder (*mais velho*)/senior (*mais velho*)/geriatric (*geriátrico*)/gerontologic (*gerontológico*)/older people (*pessoa mais velha*)/older person (*pessoa mais velha*)/fall risk (*risco de queda*)/fall (*queda*)/fall reduction (*redução de queda*)/fall prevention (*prevenção de queda*). It is important to note that the descriptor “older adult” refers to people aged 65 to 79 years old, therefore, in order not to limit searches to older young adults, indexing terms that covered individuals aged 80 years or older were also included. To list people between 60 and 65 years old, the term “middle-aged person” was included, which refers to individuals aged between 45 and 64 years old;

C (concept) - Safe mobility (*mobilidade segura*);

C (context) - Hospital Care (*Assistência hospitalar*)/Hospitals (*hospitais*).

Such terms were combined using Boolean operators OR, AND and NOT and used to develop a complete search strategy for CINAHL, which was adapted to the other databases:

(MM “Aged”) OR (MM “Aged, 80 and Over”) OR aged OR “aged patient” OR “aged people” OR “aged person” OR “middle aged” OR elderly OR “elderly patient” OR “elderly people” OR “elderly person” OR “elderly subject” OR “senior citizen” OR senium) AND (“fall risk” OR “Accidental Falls” OR “Accidental Fall” OR “Fall and Slip” OR Falling) AND “safe mobility” OR mobility AND (MM “Hospitalization”) OR (Hospitalisation OR Hospitalisations OR Hospitalizations).

The second stage consisted of searching databases, which took place in May 2022. The sources of information were BDENF/VHL, Scopus, CINAHL/EBSCO, Embase, Web of Science Core Collection, PEDro, MEDLINE/PubMed and CAPES Catalog of Theses and Dissertations.

The third stage, carried out in November 2022, referred to examination of a list of references of those articles included to select additional studies.

The study selection and evidence extraction process were carried out in a double-independent manner, with blinding through the use of free and open access Rayyan QCRI. Disagreements were resolved by a third reviewer. The selection was made by reading the titles and abstracts, followed by full reading and checking the references of articles that were the object of study in the research. An attempt was made to contact 16 authors in order to request the full texts to be made available, without success.

For extraction, an electronic form prepared by the authors was used according to previous references, and preliminarily tested, containing the article title, journal in which it was published, authors, language, year of publication, database, country of origin, objectives, study design, population, study location, constituent elements of the safe mobility concept, excerpts with the main results of interest in this review, article reference and other references found.

### Data analysis

A qualitative content analysis was carried out with an inductive approach, as recommended by Elo and Kyngas^(14-15)^, which enabled the categorization and emergence of topics of interest.

## RESULTS

Database searches revealed 521 articles, 291 in VHL, 81 in Scopus, 65 in CINAHL (EBSCO), 25 in Embase, 23 in Web of Science, 23 in PEDro and 13 in MEDLINE (PubMed). In gray literature research, material was obtained from the CAPES Theses and Dissertations Catalog.

**Figure 1:**
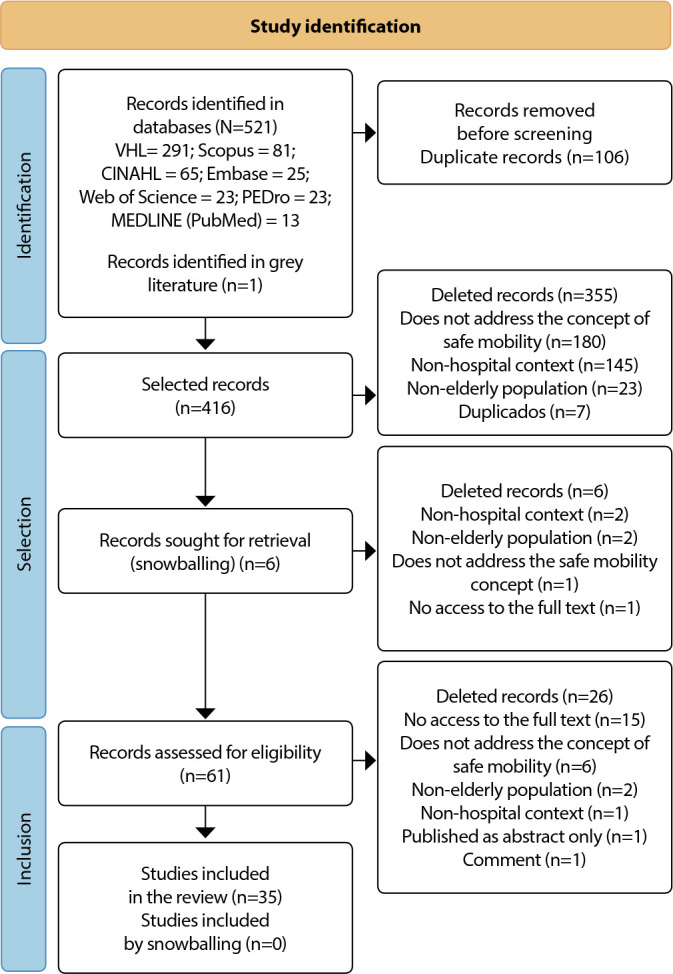
PRISMA SR flowchart for identification, selection and inclusion of studies containing constituent elements of the safe mobility concept in hospitalized older adults, Rio de Janeiro, Rio de Janeiro, Brazil, 2023

Of the 521 articles and one dissertation, 106 were duplicates and were excluded, leaving 416 for reading their respective titles and abstracts. This process led to the exclusion of 355 publications, as they did not meet the inclusion criteria, and the pre-selection of 61 articles for full reading, where six articles were recovered using the snowballing strategy. In the end, 35 works remained that suited the research objectives, as shown in Figure 1.

The texts were published in English (91.42%, n=32), Portuguese (5.71%, n=2) and German (2.85%, n=1), and produced between 1998 and 2022, with 2011 the year with the greatest relevance in the number of studies. Thus, 15 (42.85%) studies are American - 11 (31.42%) from the United States of America, two (5.71%) from Brazil and two (5.71%) from Canada; 13 (37.14%) are European - three (8.57%) from Italy, three (8.57%) from the Netherlands, three (8.57%) from Germany, two (5.71%) from England, one (2.85%) from Ireland and one (2.85%) from the United Kingdom; five are (14.28%) from Oceania - five (14.28%) from Australia; and two are (5.71%) from Asia - one (2.85%) from Israel and one (2.85%) from Singapore. With regard to methodological characteristics, 26 (74.2%) are primary studies, six (17.14%) are theoretical-conceptual studies, two (5.71%) are bibliographical reviews, one (2.85%) is secondary study. As for the approach, of the studies that specified it, eight (22.85%) are qualitative, two (5.71%) are mixed, and one (2.85%) is quantitative.

Chart 1 shows the objectives of studies according to chronological order.

**Chart 1:** Characterization of studies included in the scoping review in chronological order, Rio de Janeiro, Rio de Janeiro, Brazil, 2022

Article title		Year Country	Design Sample	Objective	Results
1	Immobility and falls^(16)^	1998 USA	Theoretical-conceptual	Not specified	Appropriate actions to prevent immobility and falls include increasing exercise and activity levels, improving the hospital environment, and decreasing the use of psychotropic medications. Bed alarms and increased supervision for high-risk patients can also help prevent falls.
2	Technology to promote safe mobility in the elderly^(17)^	2004 USA	Theoretical-conceptual	To describe new technologies designed to help prevent adverse events in the functional domain of mobility.	Key technologies to prevent falls and fall-related injuries include hipprotectors, wheelchair/scooter safety features, intelligent walkers, fall alarms, and environmental aids.
3	Why do we use physical restraints in the elderly?^(18)^	2005 Netherlands	Literature review (type of review and sample size not specified)	Answer the question “Why do we use physical restraints in older adults?”, summarizing current knowledge about the use of restraints in older adults.	With regard to prevention of falls, numerous interventions have been suggested in the literature, like floor mats, hip protectors, position alarms, motion devices, anti-slip mats, height adjustable beds, bed next to wall and multi-factorial falls risk assessment and management programs.
4	Barriers to mobility during hospitalization from the perspective of the elderly and their nurses and doctors^(19)^	2007 USA	Qualitative interviews analyzed and interpreted using a grounded theory approach. 29 participants - 10 patients > or = 75 years old, 10 nurses and 9 resident doctors	To identify barriers to mobility during hospitalization from the perspectives of older adult patients and their primary nurses and physicians, to compare and contrast the perceived barriers between these groups, and to build a conceptual model.	Content analysis identified 31 perceived barriers to increasing mobility during hospitalization. The barriers most frequently described by the three groups were symptoms (97%), especially weakness (59%), pain (55%) and fatigue (34%); have intravenous access (69%) or urinary catheter (59%); and concern about falls (79%). The lack of staff to assist with activities outside the bed was mentioned by patients (20%), nurses (70%) and doctors (67%).
5	*Der Sturz im Krankenhaus: Ein Qualitätsindikator?^(^* ^20)^	2007 Germany	Primary research. 811 “fallers” (total number of falls: 1,177) and 5,229 “non-fallers” in a geriatric hospital.	To answer the question “Are falls associated with a result of lower mobility (Barthel Index) at discharge?”.	A higher rate of falls was associated with a better outcome in two of the three mobility-related items of the Barthel Index (transfer, walk/wheelchair).
6	An elderly-centered, personalized, physiotherapy program early after cardiac surgery^(21)^	2010 Italy	Primary research. 224 consecutive patients aged 70 to 87 years followed the personalized (n = 150) or usual (n = 74) program	1) To validate our approach to personalizing the physiotherapy program by assessing the relationship between the level of frailty and patients’ functional impairment, disability and postoperative evolution; 2) To assess the effect of the personalized program on independence and mobility and compare this effect with that of our traditional training program.	At discharge, both groups had significantly improved on all measures of independence and mobility, but most of these changes (nursing needs, mobility, balance, and muscle strength) were significantly greater (P < 0.05) in the intervention group. These patients also had a significantly shorter length of stay (17.5 ± 8 vs. 21 ± 4 days, P = 0.0002), and 91% of them could be discharged in a state of substantial independence.
7	Improving care for patients with dementia hospitalized for acute somatic illness in a specialized care unit: a feasibility study^(22)^	2010 Germany	Primary research. 332 consecutively admitted patients were enrolled	To develop a segregated Special Care Unit (SCU) in a somatic hospital for patients with challenging behavior resulting from dementia and/or delirium. This pilot study assesses feasibility and patient outcomes.	Six patients were transferred to geriatric psychiatry. Two patients suffered a fall-related hip fracture. The median Barthel Index improved significantly (admission 30, discharge 45, p < 0.001), with only 8.5% of patients suffering functional loss. Wandering, aggression and agitation were significantly reduced (p < 0.001).
8	Nutritional status and associations with falls, balance, mobility and functionality during hospital admission^(23)^	2011 Australia	Observational longitudinal cohort study. Admissions to the Geriatric Assessment and Rehabilitation Unit over a period of six months were included.	To explore the associations between nutritional status, falls and selected risk factors among hospitalized older adults.	Patients assessed as malnourished were older (p<0.001) and more likely to have a poorer score on both the admission (p<0.05) and discharge (p<0.009) timed “Up and Go” test. Malnutrition was associated with reduced mobility (p<0.05).
9	Ten Ways to Improve the Care of Elderly Patients in the Hospital^(24)^	2011 USA	Theoretical-conceptual	Not specified	Ways to improve care for hospitalized older adults include: screening for geriatric syndromes such as delirium, assessing functional status and maintaining mobility, and implementing interventions that have been proven to prevent delirium, accidental falls and acute functional decline in the hospital.
10	A randomized controlled trial to prevent hospital readmissions and loss of functional ability in high risk older adults: a study protocol^(25)^	2011 Australia	Randomized controlled trial. 328 participants (82/group)	To assess innovative transitional care strategies to reduce unplanned readmissions and improve functional status, independence, and psychosocial well-being of community-dwelling older adults at risk of readmission.	Individualized exercise programs designed to improve strength, stability, coordination, endurance, mobility, and improve self-confidence with respect to activities of daily living. The exercise prescription will be developed using a team approach involving patients, caregivers, doctors, and ward nurses.
11	Predicting Habitual Physical Activity Using Coping Strategies in Older Fallers Engaged in Falls-Prevention Exercise^(26)^	2011 England	Observational study. 98 men and women	To examine physical activity in older adults who fall, applying an adaptation theory, to determine predictors of habitual physical activity.	Two coping strategies, loss-based selection and optimization, best explained the change in physical activity between baseline and follow-up.
12	Improving physical activity in older adults receiving in patient rehabilitation: a phase II feasibility study^(27)^	2012 Australia	Single-blind, randomized clinical trial (RCT) with intention-to-treat analysis 47 participants randomized to a control group (25) or intervention group (22)	To assess the feasibility of an RCT of increasing physical activity in older adults undergoing rehabilitation.	The proposed RCT of enhanced physical activity in older adults receiving rehabilitation was feasible.
13	Vestibular and Motor Contributions to Mobility: Limitations of Seniors Awaiting Discharge from Hospital Care^(28)^	2012 Canada	Experimental and correlation designs	The main objective of this article was to assess the ability of hospitalized older adults to use vestibular information to control balance. The secondary objective was to examine the influence of vestibular function and lower limb muscle strength on mobility.	Seniors awaiting discharge from hospital had impaired vestibular control of balance that was systematically associated with impaired mobility. Assessing vestibular function prior to discharge from hospital could improve discharge planning with respect to management of impairments that threaten balance and safe mobility.
14	Measure, Promote and Reward Mobility to Prevent Falls in Older Patients^(29)^	2012	Theoretical-conceptual	Not specified	A focus on maintaining and improving mobility should been couraged when an older adult becomes acutely ill and particularly vulnerable to permanently losing functional capacity during a hospitalization. More importandy, encouraging routine strength and balance training in community-dwelling older adults should be a priority.
15	What is the involvement of the nursing team in maintaining and promoting the mobility of the elderly in the hospital? A grounded theory study^(30)^	2013 England	Semi-structured interviews with 39 rehabilitation professionals and 61 hours of non-participant observation comprised the dataset	To present a grounded theory on the nursing team involvement in the process of maintaining and promoting the mobility of hospitalized older adults.	The nursing team involvement in maintaining and rehabilitating patients’ mobility was explained by the central category “care to maintain safety”. It identified how the nursing team focused primarily on preventing patient problems rather than focusing on rehabilitation goals. A number of contextual factors in the workplace meant that nursing staff had difficulty engaging in activities to support mobility maintenance and rehabilitation.
16	Activity restriction vs self-direction: hospitalised older adults’ response to fear of falling^(31)^	2014 USA	Quantitative and qualitative approach combined with medical records extraction. 41 older adults	To describe the fear of falling in hospitalized older adults and its relationship with patients’ characteristics and physical function and explore patients’ view of associated factors.	Participants described the following factors, organised by social-ecological framework, to be considered when developing alternatives to activity restriction: intrapersonal, interpersonal, environmental and policy.
17	Implementing a Comprehensive Functional Model of Care in Hospitalized Older Adults^(32)^	2014 USA	Convenience sample. 866 older adults	To develop a comprehensive model of care to promote physical function in hospitalized older adults.	Implementing a comprehensive functional model of care for hospitalized older adults had a positive impact on length of stay, 30-day readmission rate, and fall rate. The estimated cost savings associated with reducing post-intervention length of stay by 3 months was approximately $200.00.
18	*Diagnósticos e prescrições de enfermagem para idosos em situação hospitalar* ^(33)^	2015 Brazil	Qualitative research submitted to descriptive statistical analysis. 50 older adults	To identify the most frequent nursing diagnoses described by the North American Nursing Diagnosis Association among older adults in a hospital situation and propose related prescriptions for older adults in a hospital situation.	Of the older adults surveyed, 36 (72%) were diagnosed with risk of falls, manifested by the need for assistance with walking and gait disturbance. Research carried out by nurses from Minas Gerais assessed the risk factors presented for the risk for falls nursing diagnosis, observing the predominance of intrinsic factors over extrinsic ones. The most common intrinsic factors were age over 65 years (84%), use of medications (28%), difficulty walking (22%) and history of falls (22%).
19	Effectiveness of an individualized fall prevention program in a geriatric rehabilitation hospital setting: a cluster randomized study^(34)^	2015 Israel	Two-stage cluster-controlled trial conducted in five geriatric rehabilitation wards. 752 patients	To investigate the effect of an individualized fall prevention program in a geriatric rehabilitation hospital.	Although falls may occasionally have one simple explanation, they are generally the result of synergistic interactions between frailties, long-termmedical illness, acute medical illness, medications, the person’s own behavior and environmental hazards.
20	Moving forward in fall prevention: an intervention to improve balance among patients in a quasi-experimental study of hospitalized patients^(35)^	2015 Italy	Prospective quasi-experimental study. 28 patients	To investigate whether three different rehabilitation programs, such as group exercises, individual core stability or balance training intervention with a stabilometric platform, were effective in improving balance capacity in hospitalized older adult patients and evaluate whether there were differences in terms of effectiveness between these three different programs.	Participation in an exercise program can improve balance and functional mobility, which might contribute toward the reductions of falls of older adults hospitalized and the subsequent fall-related costs.
21	*Instrumento de avaliação de quedas em idosos hospitalizados (IAQI Hospitalar): enfermeiro analisando vulnerabilidade e mobilidade* ^(36)^	2016 Brazil	Exploratory and descriptive study, with a qualitative approach	To develop an instrument to assess vulnerability to falls in hospitalized older adults.	*IAQI Hospitalar* helps determine the individual profile and vulnerability of older adults so that fall prevention actions can be scheduled.
22	Comparison of posthospitalization function and community mobility in hospital mobility program and usual care patients: a randomized clinical trial^(37)^	2016 USA	Single-blind randomized clinical trial. 100 patients	To examine the effect of an in-hospital mobility program (MP) on post-hospitalization function and community mobility.	The MP intervention allowed patients to maintain pre-hospitalization community mobility, while those in the usual care group experienced clinically significant declines. Lower living space mobility is associated with increased risk of death, nursing home admission, and functional decline, suggesting that declines such as those observed in the usual care group would be of great clinical importance.
23	The effects of a high-intensity functional exercise group on clinical outcomes in hospitalised older adults: an assessor-blinded, randomised- controlled trial^(38)^	2017 Australia	Single-blind, randomized and controlled trial. 468 patients	To investigate a high-intensity functional exercises (HIFE) group in hospitalized older adults.	The results suggest that a HIFE group programme combined with individual physiotherapy may improve mobility to a similar extent to individual physiotherapy alone in hospitalised older adults. Providing physiotherapy in a group setting resulted in increased therapist efficiency. A high-intensity exercise group with individual physiotherapy may be aneffective and efficient method to provide care to older inpatients.
24	The Case for Mobility Assessment in Hospitalized Older Adults: American Geriatrics Society White Paper Executive Summary^(4)^	2018 USA	White paper supporting a greater focus on mobility as an outcome for hospitalized older adults	To assess and prevent loss of mobility in hospitalized older adults and summarize the recommendations in this white paper.	Recommendations: 1) Promote mobility assessment inacute care; 2) Advocate for more research funding; 3) Develop consensus on standardmethods to assess mobility; 4) Minimize the burden of mobility measurement; 5) Assess the feasibility of a mobility quality measure; 6) Reframe the current regulatory focus on falls in acute care to a focus on safe mobility; 7) Develop resources for acute caregivers.
25	Muscle strength is longitudinally associated with mobility in the elderly after acute hospitalization: the Hospital-ADL study^(39)^	2019 Netherlands	Multicenter, prospective, observational cohort study. 391 older adults	To determine (i) the course of mobility of older adults hospitalized in an acute situation and (ii) the association between muscle strength and the course of mobility over time controlled by influencing factors.	Muscle strength is longitudinally associated with mobility. Interventions to improve mobility including muscle strength are warranted in acute hospitalized older adults.
26	An augmented prescribed exercise program (APEP) to improve mobility in older acute medical patients - a randomized controlled pilot and feasibility study^(40)^	2019 Germany	Single-center, prospective, parallel-group, blinded, randomized (1:1) controlled pilot and feasibility study. 35 participants	To assess the feasibility of an Augmented Prescription Exercise Program (APEP) in older acute medical patients and to measure the potential effects of APEP on mobility capacity in order to assess the feasibility of a large-scale study.	This small feasibility RCT indicates that an APEP intervention may be safe and feasible in older acute medical patients. APEP may possibly induce small to moderate effects on mobility, but the clinical relevance of these effects may be limited.
27	Optimizing Function and Physical Activity in Hospitalized Older Adults to Prevent Functional Decline and Falls^(41)^	2019 USA	Theoretical-conceptual	Not specified.	Increasing physical activity of patients and decreasing falls is critically important tooptimize outcomes for patients and decrease length of hospital stays. There is no single approach that will effectively assure optimal time spent in physical activityor that a fall will not occur. Multifactorial approaches are needed that focus on in-dividual risks and challenges within each individual.
28	Predictors of physical activity in older adults at the start of an emergency hospital stay: a prospective cohort study^(42)^	2020 United Kingdom	Secondary analysis of a prospective repeated measures cohort study. 62 participants	To investigate predictors of in-hospital activity during a 24-hour period in the first 48 hours of hospital admission in older adults.	Physical activity, particularly in the acute phase of hospitalisation, is very low in older adults. The association between illness severity and physical activity may be explained by symptoms of acute illness being barriers to activity.
29	Promoting mobility among hospitalized elderly: an exploratory study on the perceptions of patients, caregivers and nurses^(43)^	2020 Singapore	Descriptive qualitative study with a purposeful and convenience sampling approach. 14 patients, six caregivers and ten nurses	To explore the perceptions of patients and their caregivers as well as nurses on promoting mobility among hospitalized older adult patients.	Recognition of the importance of mobility as well as the detrimental effects of prolonged bed rest during hospitalization is an essential first step toward developing successful interventions to promote mobility in the Asian context. Improvements need to be made to help overcome the various barriers and challenges in the mobilization of older patients in acute care settings. Nurses and other care team members can help to increase the confidence of patients and among family caregivers (in providing assistance during mobility) by role modelling and provision of walking aids as well as risk-based education.
30	Factors associated with walking in hospitalized elderly^(44)^	2020 Ireland	Cohort study. 154 participants	To identify patient characteristics upon admission and daily events during hospitalization that could influence the walking activity of older adult patients during hospitalization.	Daily step count fluctuated, suggesting considerable scope for intervention. Devices or treatments that hinder walking should be reviewed daily and walking activity should become a clinical priority. Admission physical performance may identify vulnerable patients.
31	Assisted Walking Program on Walking Ability in In-Hospital Geriatric Patients: A Randomized Trial^(45)^	2021 Italy	RCT. 387 patients	To assess whether an individualized assisted walking program for hospitalized older adult patients could improve walking capacity compared to usual geriatric care and rehabilitation.	Baseline characteristics were similar between intervention and control groups. The intervention group, relative to the control group, had significantly improved walking ability at discharge (P < .001). There were no statistically significant differences between the groups in terms of in-hospital adverse events. No adverse effects were detected.
32	Reimagining Injurious Falls and Safe Mobility^(2)^	2021 USA	Theoretical-conceptual	To propose a new approach to reducing falls with injuries in older adults based on evidence-based protocols known to positively impact older adults’ health.	ERA - Electronic health record integration, Risk factors that matter, Assessment and care plans - allows nurses to use a validated fall risk assessment tool to reframe fall risk factors as part of a comprehensive care plan, and to map modifiable risk factors to interventions that address the underlying causes of falls and promote safer mobility. The ERA approach can help nurses use their time more effectively by focusing on targeted actions that improve patient outcomes, working in coordination with an interprofessional, cross-continuum care team.
33	Effects of General Physical Activity Promoting Interventions on Functional Outcomes in Patients Hospitalized over 48 Hours: A Systematic Review and Meta-Analysis of Randomized Controlled Trials^(46)^	2021 Netherlands	Systematic review study: five electronic databases were searched for RCT. For results reported in two or more studies, meta-analysis was performed to test differences between groups	To assess the effect of general physical activity, promoting interventions on functional and hospital outcomes in patients hospitalized for more than 48 hours.	Overall, we found no conclusive evidence on the effect of general physical activity promoting interventions on functional outcomes.
34	Pilot testing of nurse led multimodal intervention for falls prevention^(47)^	2022 USA	Preand post-test pilot study in a single group. 70 patients	To examine the effect of a nurse-led multimodal intervention on levels of fall risk awareness, self-efficacy, and involvement in fall prevention among hospitalized adults.	There were significant differences [pre-test (M= 19.02, SD=1.152) and post-test (M= 21.71, SD=0.527); t (17.355) = 58,p.001] on level of fall risk awareness in fall prevention. There were no significantfindings for fall self-efficacy and engagement. Study findings suggested that the higher the fall self-efficacy, the higher the engagement. Future research is needed to examine self-efficacy and engagement for fall prevention in larger diverse cohorts of hospitalized older adults.
35	Effect of a Ward-Based Program on Hospital-Associated Complications and Length of Stay for Older Inpatients The Cluster Randomized CHERISH Trial^(48)^	2022 Australia	Pragmatic cluster randomized trial. 539 patients	To implement and assess a ward-based improvement program (“Eat Walk Engage”) to more consistently provide older adult-friendly principles of care to older adults in acute patient ward situations.	Eat Walk Engage did not reduce the composite primary outcome of any HAC-OP, which affected almost half of older inpatients, although there was a significant reduction in delirium.

**Figure 2:**
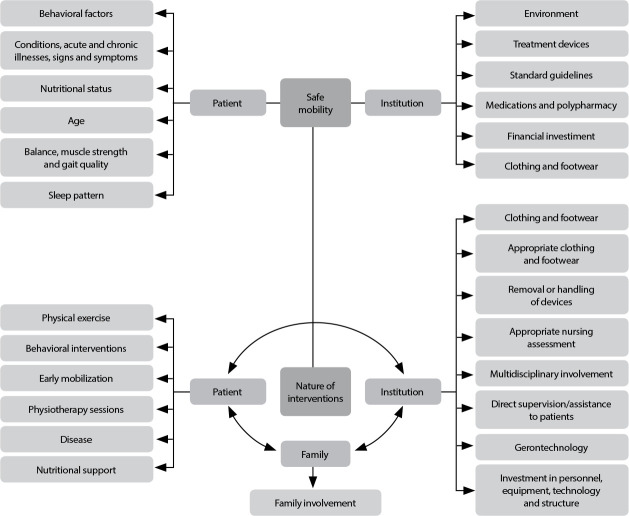
Constituent elements of the safe mobility concept in hospitalized older adults related to patients, the institution and the nature of the interventions, Rio de Janeiro, Rio de Janeiro, Brazil, 2023

None of the studies presented a clear safe mobility concept, however the concern that mobility must be promoted in order to guarantee patient safety, comfort, quality of life and prevent high dependence stands out^(43)^. Although the concept is not precise, constitutive elements of the concept were identified, which are related to patients, the institution and the nature of the interventions, as shown in Figure 2.

## DISCUSSION

The extent of available evidence mapped in this scoping review showed that intrinsic factors related to patients are those cited most frequently with regard to promoting mobility. Behavioral factors^(2,16,19,21,27,29,31,33-36,39-40,43,46-47)^ are the most prevalent, being mentioned in around 43% of the texts (n =15). Among them, sedentary behavior^(31)^ and inactivity^(46)^, social isolation and loneliness^(2)^, lack of motivation^(46)^, depressive symptoms^(31,39,41)^, fear of falling^(21,31,37,39,41,44)^ and concerns about the injuries that falls can cause^(44)^, in addition to beliefs and perspectives about mobility^(37)^, such as associating the idea of being hospitalized with staying in bed to recover health or that it is a rule of the institution that patients must remain in their rooms, emerge as factors that limit older adults’ confidence, becoming obstacles to mobility promotion.

In this regard, achieving safe mobility presupposes assessing behavioral factors, proposing multifaceted interventions, encouraging social interaction^(29,33,47)^, such as group meals or activities during the day, together and outside the room, and guidelines verbal and written messages to patients^(24,30-32,43)^ tend to motivate them^(41,45)^ and encourage them^(30-31,45)^. As a coping strategy, progressive goals can be set^(41)^, initially encouraging them to perform their basic activities of daily living independently^(25)^. Furthermore, multidisciplinary involvement is important, since patients who are repeatedly warned about leaving their beds evoke aggressive behavior, in addition to hindering initiatives by older adults^(22)^.

Another set of factors associated with safe mobility are acute and chronic conditions and illnesses^(16,19,22,28,34,36,41,43,46-47)^. Although one study revealed that there was no influence of comorbidities on the relationship between muscular strength and mobility^(39)^, the other authors present conditions (fragility)^(34)^, pathologies (urinary incontinence, visual impairment^(28,37,41-42,44,46)^, anemia^(41)^, delirium and dementia^(22,32,36,39,41)^, among others) as well as the signs and symptoms arising from them^(16,19,28,34,36,41-42,46-47)^ (shortness of breath^(46)^, respiratory and cardiovascular instability^(42)^, fatigue^(37,39,42,44)^, weakness^(37,44)^ and pain^(37,40-42,44,46)^), lead to disability or increase the risk of falling. Therefore, for safe mobility, it is necessary to recognize the impact of diseases and their appropriate management, considering, however, two other relevant factors: treatment devices^(5,31,37,41-42,44)^ and medications and polypharmacy^(16,34,42)^.

Intravenous catheter and equipment^(5,30,37,41,43)^, indwelling bladder catheter^(5,31,37,41)^, oxygen in the gas network^(41,44)^, monitoring^(41)^ and drains^(46)^ are treatment devices frequently used in hospital units, with a view to recovering health, which, however, impair mobility. Therefore, it is proposed the early removal of intravenous catheters, the momentary interruption of slow infusions or use of an IV stand with wheels, so that it can be transported safely^(44)^, and the provision of supplemental oxygen through small portable cylinders^(44)^.

Regarding drug therapy^(16,34,41)^, this must be assessed, paying particular attention to polypharmacy^(41)^ and the prescription of sedatives, psychotropics, diuretics and hypotensives^(24)^, drugs whose effects make older adults more vulnerable to the fall^(2,41)^. The greater the number of medications in use, the lower the level of self-efficacy and engagement in preventing falls^(47)^. Therefore, one must consider the complexity of managing falls prevention and safe mobility of hospitalized older adults, which add to the conditions of senescence and senility, and which, if not proposed appropriately, could worsen patients’ condition.

Muscular strength^(23,36,38)^, gait quality^(36)^ and balance^(27-28,36)^ are also essential prerequisites for safe mobility. Compromised muscle strength and balance culminate in postural instability and, therefore, a predisposition to falls^(36)^. Therefore, it is proposed to carry out exercises^(23,39-40)^ that improve strength, core stability, coordination, resistance^(38)^ and body mechanics, developing confidence^(25)^, such as structured exercise programs^(24)^ individual or group, balance^(26)^ and training on a stabilometric platform^(27)^, in addition to sitting and standing exercises, progressing to changing weight, in a fixed place and then walking^(37)^, strengthening with weight^(26)^, walking plan^(26)^ and joint exercises^(33)^.

To carry out the exercises, two questions must be considered. The first is nutritional support. While one study revealed that Body Mass Index and nutritional status did not influence the relationship between muscular strength and mobility^(39)^; others, on the other hand, revealed that obesity and malnutrition^(22,36,40-42)^ are associated with muscle loss, worse physical function and consequent risk of falling, and, therefore, mobilization must be scrupulous^(42)^. In this regard, particularly in exercises with resistance and strength training, nutritional support is essential so that there is no weight loss^(22)^.

The second issue is that exercises are planned considering non-maleficence. Promoting physical activity is a simple and non-invasive intervention, with the potential to improve mobility^(26)^; however, for it to be safe, it must be dosed so that it does not cause symptoms such as dyspnea, weakness and fatigue^(31)^. Safe physical activity prevents the loss of function and physiological reserve due to immobility and accelerates the restoration of functions lost due to acute illnesses^(16,46)^, even contributing to post-discharge community mobility^(18)^. It is even recommended that mobilization be started early^(21,24,32,45,47)^, from the moment of hospitalization, based on an initial risk assessment^(30)^ so that pre-hospitalization is maintained^(16)^, already intending to plan discharge^(28,40)^.

However, there are divergences in the literature regarding the designation of the professional in charge of promoting safe mobility, particularly in more dependent patients who require the support of a professional^(37)^. There is a general consensus that the interprofessional and continuous care approach is beneficial for both the system and the patients^(2,16,22,29,42,48)^; however, the texts highlight physiotherapists’ and nurses’ leading role.

Physiotherapists promote sessions with assessment and treatments that maximize mobility and independence through the prescription and delivery of exercises, contributing to older adults’ confidence as they progress and follow-up^(21,38,40)^. It is understood, however, from the readings, that these are specific interventions.

Nurses greatly close to patients are essential to directly assist and supervise patients in promoting mobility (moving, getting out of bed, walking)^(30,33,44)^. However, some issues highlighted by nurses need to be highlighted: 1) Lack of staff and overworked nursing^(4,42)^ - there is a need for greater presence of physiotherapy^(37)^; 2) Nurses do not feel qualified to provide physical assistance and assess it appropriately^(42)^ (This is one of the reasons why walking is not routinely encouraged, causing, in turn, an excessive dependence on physiotherapy^(4)^ as well as misleading guidance, such as ordered bed rest^(44)^, even for patients who do not need assistance. Furthermore, many nurses do not understand the validity, reliability and usefulness of mobility assessment measures^(4)^, just as they do not there are standardized and validated processes to encourage older adults’ safe mobility during hospitalization^(4)^); 3) Some nurses do not identify mobility promotion as their responsibility, not collaborating with physiotherapists and postponing guidance^(4)^.

Finally, it is understood that nursing assists in the process of physical and personal care, supporting movement, transfer and basic activities, which require supervision^(30)^. Nursing assessment is important in the identification of geriatric syndromes^(24)^ as well as in assessment and recognition of fall risk factors, which must be included in a care plan, in addition to risks related to the environment^(2)^. Physiotherapy, in turn, specializes in movement, handling and rehabilitation, and is responsible for these functions^(30)^.

Still regarding the challenges to promoting safe mobility, it is important to highlight the lack of financial investments and equipment, such as walking aids^(4,31,37,44,46)^, which allow greater freedom and safety^(36)^. Another possibility is gerontechnology resources, such as gait belts^(19)^, hip protectors^(17-18)^, smart walker^(17-18)^, fall alarms^(17-18)^, anti-slip mat^(18)^ and movement sensors^(6)^. It is interesting that the nursing team is at the forefront of selecting and testing equipment, integrating new technologies into existing infrastructure so that adverse effects related to mobility are eliminated or mitigated, which favors patient treatment, rehabilitation and safety^(17)^.

Furthermore, it is important that the environment is improved^(17,34,43)^, such as adequate bed and furniture height^(24,27,31)^; that the ward structure makes it possible to view several patients at the same time^(41)^, with open corridors that facilitate walking^(41)^, high-impact carpets and padded floors that minimize the risk of injury^(24,29,41)^; that there is adequate lighting in corridors and rooms^(22,32)^; and that the hospital is signposted and maps are made available so that patients can find their way around the institution^(36,48)^.

Finally, appropriate clothing^(19)^ and footwear^(22,32)^ must be provided, attention should be paid to sleep quality^(42,44)^, length of stay^(20)^, age, as advancing age contributes for a greater risk of falling^(33)^, and integrating the family into the care of older adults, making them facilitators and defenders^(31)^ of safe mobility, supporting and reducing negative feelings^(2,22)^.

Therefore, mapping the evidence showed that safe mobility is related to the nature of the interventions and non-modifiable and modifiable risk factors related to patients, with modifiable risk factors being subject to intervention. Aspects related to the institution include professional training and qualification, adequate staffing, investment in equipment, technologies and structural reforms, in addition to offering appropriate clothing and footwear that converges with the idea.

During the mapping of studies, it was observed that mobility promotion for older adults was valued as well as early mobilization; however, few studies were concerned with studying how to carry it out safely. In this sense, this gap indicates the need for more studies to be carried out that highlight patient safety.

### Study limitations

The limitations of this study involve limiting the approaches of studies on the dimensions of older adults’ lives that can influence safe mobility and risks of accidents due to falls, such as cognitive, mental and emotional aspects, in addition to not delving deeper into drug therapy. Furthermore, limitations may be related to the search in a simple number of gray literature sources.

### Contributions to health

The review’s contribution to health points to changes in the perception of falls prevention in hospitalized older adults over recent years. A study carried out in 2007 argues that, until data on successful strategies were available, minimizing mobility could remain the standard solution for preventing falls^(19)^, whereas a 2021 study understands that preserving mobility and independence requires some risks of falls^(2)^. The most successful interventions tested are related to muscle strengthening and balance exercise promotion^(27-29,32,35,38-39,41,45)^.

## FINAL CONSIDERATIONS

Evidence regarding the constituent elements of safe mobility supports that these are related to patients (behavioral factors, conditions, acute and chronic diseases, signs and symptoms, nutritional status, age, balance, muscle strength and quality of gait and sleep pattern), the institution (environmental risks, treatment devices, mistaken guidelines, medications and polypharmacy, resources, clothing and footwear) and the nature of the interventions (related to the family, the patient and the institution), assuming that greater possibilities for intercession are related to the last and involve multiple dimensions. Moreover, safe mobility is an expression of hospital units’ ability to guarantee care and protection from fall accidents for older adults. The present review showed, however, that the resources for preparing the environment and health professionals to deal with older adults’ specific demands are insufficient. Finally, it is important to suggest carrying out a concept analysis of the term “safe mobility”.
