# Supplementary material for: Nursing care for hospitalized older adults - fall accidents versus safe mobility: a scoping review
Source: Rev Bras Enferm. 2024 Jul 19;77(2):e20230180. doi: 10.1590/0034-7167-2023-0180 (PMC11259443; doi:10.1590/0034-7167-2023-0180)
Supplement: Translation

# Translation: Cuidado de enfermagem à pessoa idosa hospitalizada - acidentes por quedas *versus* mobilidade segura: revisão de escopo

**DOI:** 10.1590/0034-7167-2023-0180pt

**Authors:** Esther Mourão Nicoli, Frances Valéria Costa e Silva, Luciana Guimarães Assad, Camila Castanho Cardinelli, Raquel Azevedo Alves, Samara Gonçalves de Oliveira

**Affiliations:** I Universidade do Estado do Rio de Janeiro. Rio de Janeiro, Rio de Janeiro, Brasil

**Keywords:** Acidentes por Quedas, Cuidado de Enfermagem, Limitação da Mobilidade, Idoso, Assistência Hospitalar

## Abstract

**Objetivos:**

mapear os elementos constitutivos do conceito de mobilidade segura presentes no cuidado hospitalar a pessoas idosas.

**Métodos:**

revisão de escopo de 35 artigos buscados em bases de dados e literatura cinzenta - BDENF/BVS, Scopus, CINAHL/EBSCO, Embase, *Web of Science*, PEDro, MEDLINE/PubMed e Catálogo de Teses e Dissertações da CAPES. Nenhum recorte temporal e de idioma foi estabelecido.

**Resultados:**

nenhum dos estudos apresentou o conceito claro de mobilidade segura, entretanto seus elementos constitutivos envolvem fatores relacionados ao paciente (fatores comportamentais, condições, doenças, sinais e sintomas, estado nutricional, idade, equilíbrio, força, qualidade da marcha, sono), à instituição (ambiente, dispositivos para tratamento, orientações, medicamentos e polifarmácia, recursos materiais e humanos e vestimentas/calçados) e à natureza das intervenções (relacionadas ao paciente, à instituição e à família).

**Considerações Finais:**

os elementos constitutivos de mobilidade segura expressam a capacidade das unidades hospitalares em garantir cuidado e proteção de acidentes por quedas a pessoas idosas hospitalizadas.

## INTRODUÇÃO

A ocorrência de quedas em pessoas idosas, apesar dos contínuos esforços da enfermagem para reduzi-las, ainda representa um dos incidentes mais importantes em ambientes hospitalares, ponderando sua frequência, impactos econômicos e consequências^(1)^.

Tal situação decorre dos programas e protocolos de prevenção de quedas implementados, que tendem a simplificar o evento e sobrevalorizar a prevenção em si, negligenciando a avaliação do risco de quedas em um plano de cuidados^(2)^. Por conseguinte, observa-se uma maior ênfase no somatório e registro da pontuação obtida por meio da aplicação de escalas preditivas de quedas enquanto menos tempo é dedicado à proposição de intervenções que abordem fatores de risco modificáveis^(2)^.

Durante as últimas quatro décadas, as práticas de enfermagem relacionadas à prevenção de quedas não sofreram alterações significativas^(3)^. Habitualmente, as medidas adotadas concentram-se em reduzir a mobilidade do paciente ou melhorar a segurança do ambiente, sem considerar que os fatores intrínsecos são, na verdade, os principais precursores das quedas^(2)^.

No entanto, intervenções que limitam a mobilidade, como pulseiras de sinalização, recomendações de repouso no leito ou a redução do tempo de uso de toaletes, apesar de oferecerem alguma proteção contra quedas, podem suscitar declínio funcional e o desenvolvimento de síndromes geriátricas, como instabilidade postural, imobilidade e iatrogenia^(4)^. Ademais, tais intervenções, restritivas, aumentam o tempo de internamento, o risco de re-hospitalização e a probabilidade de complicações que, por sua vez, exacerbam o risco de quedas futuras^(4)^.

Durante o período de hospitalização, os pacientes permanecem sentados ou deitados em média de 87% a 100% do tempo^(5)^. Por conseguinte, em pessoas idosas, mesmo que saudáveis, dez dias de repouso no leito equivalem a uma redução de aproximadamente 12% da capacidade aeróbica e 16% da força dos músculos extensores do joelho, o que impacta um declínio significativo da capacidade funcional^(2)^.

Nesse contexto, o conceito “mobilidade segura” expressa uma transição de perspectiva, avaliação e reconhecimento dos fatores de risco de quedas^(2)^. Há, portanto, um destaque para os fatores intrínsecos, sem, por sua vez, desconsiderar protocolos, escalas preditivas e fatores extrínsecos, também importantes, integrando as evidências ao plano de cuidados de abordagem inter e multiprofissional^(2)^.

A mobilidade segura se faz centrada no paciente, valorizando sua individualidade, preferências, necessidades e valores^(6)^. Destarte, a pessoa idosa é devidamente informada e consultada, para que, então, sejam tomadas decisões compartilhadas que considerem sua vontade, questões técnicas e evidências científicas^(6)^. Quando a pessoa idosa participa ativamente do planejamento de seus cuidados, pode tomar consciência dos riscos e de sua própria condição, o que, por sua vez, a motiva a adotar comportamentos de mobilidade segura^(6)^.

Essa abordagem implica, portanto, um maior pensamento crítico^(2)^. É imprescindível avaliar cautelosamente as orientações de restrição ao leito, além de considerar que a preservação da mobilidade pode envolver algum risco de queda^(2)^. Todavia, um plano de mobilidade realista pode ser mais benéfico para o bem-estar da pessoa idosa do que o esfoço de evitar quedas a qualquer custo^(2)^.

Os enfermeiros, enquanto gestores do cuidado e agentes influentes nesse contexto, desempenham um papel fundamental na forma de prevenir de quedas^(7)^. Eles são responsáveis pela introdução e disseminação de intervenções que considerem as necessidades individuais de cada pessoa idosa, visando seu bem-estar, segurança e autonomia^(7)^. Nessa visão, aclarar a compreensão dos elementos constitutivos do conceito “mobilidade segura” amplia a possibilidade de construir intervenções de enfermagem alinhadas a esse objetivo, sendo essa uma lacuna de conhecimento.

Em janeiro de 2022, foi conduzida uma busca preliminar nas bases de dados PubMed, CINAHL e JBI, que revelou uma escassez de estudos sobre o tema. Isso motivou a proposta de elaboração de uma revisão de escopo no intuito de mapear documentos que tratam do termo “mobilidade segura” na literatura^(8)^. Tal estudo fortalece o movimento de produção científica no universo temático assinalado, uma vez que a revisão de escopo possibilita identificar as lacunas na literatura, esclarecer os conceitos e resumir as descobertas, além de sistematizar e disseminar os achados que podem contribuir para as práticas, políticas e pesquisas^(8)^.

## OBJETIVOS

Mapear os elementos constitutivos da mobilidade segura presentes no cuidado hospitalar a pessoas idosas.

## MÉTODOS

### Delineamento do estudo

Trata-se de estudo de revisão de escopo orientado conforme diretrizes do JBI, organização internacional de pesquisa que orienta revisões sistemáticas^(9)^. Inicialmente, foi estabelecida a questão de revisão, estruturada pelo acrônimo PCC - P (população/participante), C (conceito) e C (contexto)^(10)^: quais os elementos constitutivos do conceito de mobilidade segura em pessoas idosas hospitalizadas com risco de queda presentes nas publicações nacionais e internacionais?

O protocolo de revisão foi registrado no *Open Science Framework* (OSF), sob o DOI 10.17605/OSF.IO/EDHF6, e posteriormente publicado na *Online Brazilian Journal of Nursing* (OBJN)^(11)^.

Prezando pela qualidade e lisura da redação desta produção, foram seguidas as orientações do *checklist Preferred Reporting Items for Systematic reviews and Meta- Analyses extension for Scoping Reviews* (PRISMA-ScR)^(12)^.

### Critérios de elegibilidade

Os critérios de elegibilidade estão atrelados à estrutura do acrônimo PCC. Para população/participante, foram contempladas pessoas idosas - indivíduos com 60 anos ou mais, conforme classificação do Estatuto da Pessoa Idosa (Lei 14.423, de 2022)^(13)^- , de ambos os sexos. Para o conceito, foram integrados estudos que definam, relatem ou disponham sobre a mobilidade segura - abordagens pertinentes que auxiliem ou encorajem pessoas idosas a se movimentarem diariamente com segurança, visando à preservação da capacidade funcional - e os fatores associados à promoção desta. Foram excluídos os estudos que versam sobre a mobilidade urbana. Para o contexto, foram incorporados estudos que envolvam o cuidado à pessoa idosa hospitalizada, em múltiplas circunstâncias (clínica, cirúrgica, dentre outras), abrangendo hospitais públicos ou privados, de pequeno, médio ou grande porte, de ensino, geral, especializado, urbano ou rural.

A revisão considerou estudos de pesquisa primária, revisões sistemáticas, metassínteses e relatos de caso, com delineamento quantitativo ou qualitativo. Ademais, foram incluídos relatórios, textos institucionais com relevância na área da geriatria/gerontologia, livros e *guidelines* publicados nas fontes indexadas consultadas ou na literatura cinzenta. Foram excluídos os artigos publicados apenas como resumo, cartas ao editor e comentários. Nenhum recorte temporal e de idioma foi estabelecido.

### Coleta de dados

Para a coleta de dados, foi desenvolvida a estratégia de busca em três etapas. A etapa inicial, realizada em abril de 2022, consistiu na identificação dos termos de busca, e, para tal, consultaram-se os vocábulos controlados da área da saúde DeCS (Descritores em Ciências da Saúde), MeSH (*Medical Subjective Headings*) e Emtree (*Embase Subject Headings*). Foram incluídos os seguintes termos:

P (população/participante) - *middle aged* (pessoa de meia-idade)/*aged* (idoso)/*old people* (pessoa idosa)*/old person* (pessoa idosa)*/elderly* (idoso)*/Elder* (mais velho)*/senior* (mais velho)/*geriatric* (geriátrico)*/gerontologic* (gerontológico)/*older people* (pessoa mais velha)/*older person* (pessoa mais velha)/*fall risk* (risco de queda)*/fall* (queda)/*fall reduction* (redução de queda)*/fall prevention* (prevenção de queda). É importante notar que o descritor “idoso” se refere a pessoas de 65 a 79 anos de idade, portanto, a fim de não limitar as buscas a pessoas idosas jovens, também foram incluídos termos de indexação que abrangessem indivíduos de 80 anos ou mais. Para elencar pessoas entre 60 e 65 anos, foi incluído o termo “pessoa de meia-idade”, que se refere a indivíduos com idades entre 45 e 64 anos;

C (conceito) *- Safe mobility* (mobilidade segura);

C (contexto) - *Hospital Care* (Assistência hospitalar)/*Hospitals* (hospitais).

Tais termos foram combinados através dos operadores booleanos *OR, AND* e *NOT* e utilizados para o desenvolvimento de uma estratégia de pesquisa completa para a CINAHL, que foi adaptado às demais bases:

(MM “Aged”) OR (MM “Aged, 80 and Over”) OR aged OR “aged patient” OR “aged people” OR “aged person” OR “middle aged” OR elderly OR “elderly patient” OR “elderly people” OR “elderly person” OR “elderly subject” OR “senior citizen” OR senium) AND (“fall risk” OR “Accidental Falls” OR “Accidental Fall” OR “Fall and Slip” OR Falling) AND “safe mobility” OR mobility AND (MM “Hospitalization”) OR (Hospitalisation OR Hospitalisations OR Hospitalizations).

A segunda etapa consistiu na busca em bases de dados, que ocorreu em maio de 2022. As fontes de informação foram BDENF/BVS, Scopus, CINAHL/EBSCO, Embase, *Web of Science Core Collection*, PEDro, MEDLINE/PubMed e Catálogo de Teses e Dissertações da CAPES.

A terceira etapa, realizada em novembro de 2022, referiu-se ao exame da lista de referências daqueles artigos incluídos para a seleção de estudos adicionais.

O processo de seleção de estudos e extração de evidências foi realizado de modo duplo-independente, com cegamento através da utilização do *software Rayyan QCRI,* de acesso livre e gratuito. Os dissensos foram sanados por um terceiro revisor. A seleção se deu pela leitura dos títulos e resumos, sucedida pela leitura integral e verificação das referências dos artigos que foram objeto de estudo da pesquisa. Foi realizada tentativa de contato com 16 autores, a fim de solicitar a disponibilização dos textos na íntegra, sem sucesso.

Para extração, foi utilizado um formulário eletrônico elaborado pelas autoras conforme referências prévias, e preliminarmente testado, contendo título do artigo, revista na qual foi publicado, autores, idioma, ano de publicação, base de dados, país de origem, objetivos, desenho do estudo, população, local do estudo, elementos constitutivos do conceito de mobilidade segura, trechos com os principais resultados de interesse desta revisão, referência do artigo e outras referências encontradas.

### Análise dos dados

Realizou-se uma análise qualitativa do conteúdo com abordagem indutiva, conforme recomendações de Elo e Kyngas^(14-15)^, que possibilitou a categorização e emersão de tópicos de interesse.

## RESULTADOS

As buscas nas bases de dados revelaram 521 artigos, sendo 291 na BVS, 81 na Scopus, 65 na CINAHL (EBSCO), 25 na Embase, 23 na *Web of Science*, 23 na PEDro e 13 na MEDLINE (PubMed). Na pesquisa da literatura cinzenta, um material foi obtido no Catálogo de Teses e Dissertações da CAPES.

Dos 521 artigos e uma dissertação, 106 estavam duplicados e foram excluídos, restando 416 para leitura dos respectivos títulos e resumos. Esse processo conduziu à exclusão de 355 publicações, por não se adequarem aos critérios de inclusão, e à pré-seleção de 61 artigos para leitura na íntegra, onde seis artigos foram recuperados pela estratégia *snowballing*. Ao final, restaram 35 trabalhos que se adequaram aos objetivos da pesquisa, conforme Figura 1.

**Figura 1:** Fluxograma PRISMA SR de identificação, seleção e inclusão dos estudos contendo elementos constitutivos do conceito de mobilidade segura em pessoas idosas hospitalizadas, Rio de Janeiro, Rio de Janeiro, Brasil, 2023

Os textos foram publicados em inglês (91,42%, n=32), português (5,71%, n=2) e alemão (2,85%, n=1), e produzidos entre 1998 e 2022, sendo 2011 o ano com maior relevância do número de produções. Assim, 15 (42,85%) estudos são americanos - 11 (31,42%) dos Estados Unidos da América, dois (5,71%) do Brasil e dois (5,71%) do Canadá; 13 (37,14%) são europeus - três (8,57%) da Itália, três (8,57%) dos Países Baixos, três (8,57%) da Alemanha, dois (5,71%) da Inglaterra, um (2,85%) da Irlanda e um (2,85%) do Reino Unido; cinco são (14,28%) da Oceania - cinco (14,28%) da Austrália; e dois são (5,71%) da Ásia - um (2,85%) de Israel e um (2,85%) de Singapura. No que se refere às características metodológicas, 26 (74,2%) são estudos primários, seis (17,14%), teórico-conceitual, dois (5,71%), revisões bibliográficas, um (2,85%), estudo secundário. Quanto à abordagem, dos estudos que a especificaram, oito (22,85%) são qualitativos, dois (5,71%), mistos, e um (2,85%), quantitativo.

Os objetivos dos estudos podem ser observados no Quadro 1, conforme ordem cronológica.

**Quadro 1:** Caracterização dos estudos incluídos na revisão de escopo em ordem cronológica, Rio de Janeiro, Rio de Janeiro, Brasil, 2022

Título do artigo		Ano País	Delineamento Amostra	Objetivo	Resultados
1	*Immobility and falls* ^(16)^	1998 EUA	Teórico-conceitual	Não especificado	As ações adequadas para prevenir a imobilidade e as quedas incluem aumentar os níveis de exercício e atividade, melhorar o ambiente hospitalar e diminuir o uso de medicamentos psicotrópicos. Alarmes nas camas e maior supervisão para pacientes de alto risco também podem ajudar a prevenir quedas.
2	*Technology to promote safe mobility in the elderly* ^(17)^	2004 EUA	Teórico-conceitual	Descrever novas tecnologias projetadas para ajudar a prevenir eventos adversos no domínio funcional da mobilidade.	As principais tecnologias para prevenir quedas e lesões relacionadas a quedas incluem protetores de quadril, recursos de segurança para cadeiras de rodas/cadeiras de rodas elétricas, andadores inteligentes, alarmes de queda e auxílios ambientais.
3	*Why do we use physical restraints in the elderly?* ^(18)^	2005 Países Baixos	Revisão de literatura (não especificados o tipo de revisão, bem como o tamanho da amostra)	Responder à pergunta “Por que usamos restrições físicas em idosos?”, resumindo o conhecimento atual sobre o uso de restrições em idosos.	No que diz respeito à prevenção de quedas, inúmeras intervenções têm sido sugeridas na literatura, como tapetes, protetores de quadril, alarmes de posição, dispositivos de movimento, tapetes antiderrapantes, camas com altura ajustável, cama próxima à parede e avaliação multifatorial de risco de quedas e programas de gestão.
4	*Barriers to mobility during hospitalization from the perspective of the elderly and their nurses and doctors* ^(19)^	2007 EUA	Entrevistas qualitativas analisadas e interpretadas usando uma abordagem de teoria fundamentada nos dados. 29 participantes - 10 pacientes > ou = 75 anos, 10 enfermeiros e 9 médicos residentes	Identificar barreiras à mobilidade durante a hospitalização a partir das perspectivas dos pacientes idosos e dos seus enfermeiros e médicos primários, comparar e contrastar as barreiras percebidas entre estes grupos, e construir um modelo conceptual.	A análise de conteúdo identificou 31 barreiras percebidas para o aumento da mobilidade durante a hospitalização. As barreiras mais frequentemente descritas pelos três grupos foram sintomas (97%), especialmente fraqueza (59%), dor (55%) e fadiga (34%); possuir acesso intravenoso (69%) ou cateter urinário (59%); e preocupação com quedas (79%). A falta de pessoal para auxiliar nas atividades fora do leito foi mencionada pelos pacientes (20%), enfermeiros (70%) e médicos (67%).
5	*Der Sturz im Krankenhaus: Ein Qualitätsindikator?* ^(20)^	2007 Alemanha	Pesquisa primária. 811 “caidores” (número total de quedas: 1.177) e 5.229 “não caidores” em um hospital geriátrico.	Responder à pergunta “As quedas estão associadas a um resultado de menor mobilidade (Índice de Barthel) na alta?”.	Uma maior taxa de quedas foi associada a um melhor resultado em dois dos três itens relacionados à mobilidade do Índice de Barthel (transferência, caminhada/cadeira de rodas).
6	*An elderly-centered, personalized, physiotherapy program early after cardiac surgery* ^(21)^	2010 Itália	Pesquisa primária. 224 pacientes consecutivos com idades entre 70 e 87 anos seguiram o programa personalizado (n = 150) ou habitual (n = 74)	1) Validar nossa abordagem para personalização do programa de fisioterapia avaliando a relação entre o nível de fragilidade e o comprometimento funcional, incapacidade e evolução pós-operatória do paciente; 2) Avaliar o efeito do programa personalizado na independência e mobilidade e comparar este efeito com o do nosso programa de treino tradicional.	Na alta, ambos os grupos melhoraram significativamente em todas as medidas de independência e mobilidade, mas a maioria dessas alterações (necessidades de enfermagem, mobilidade, equilíbrio e força muscular) foram significativamente maiores (P <0,05) no grupo de intervenção. Esses pacientes também tiveram um tempo de internação significativamente menor (17,5 ± 8 vs. 21 ± 4 dias, P = 0,0002), e 91% deles puderam receber alta em estado de independência substancial.
7	*Improving care for patients with dementia hospitalized for acute somatic illness in a specialized care unit: a feasibility study* ^(22)^	2010 Alemanha	Pesquisa primária. 332 pacientes admitidos consecutivamente foram inscritos	Desenvolver uma Unidade de Cuidados Especiais (UCE) segregada em um hospital somático para pacientes com comportamento desafiador resultante de demência e/ou *delirium*. Este estudo piloto avalia a viabilidade e os resultados do paciente.	Seis pacientes foram transferidos para psiquiatria geriátrica. Dois pacientes sofreram fratura de quadril relacionada à queda. A mediana do Índice de Barthel melhorou significativamente (admissão 30, alta 45, p < 0,001), com apenas 8,5% dos pacientes sofrendo perda funcional. A deambulação, a agressividade e a agitação foram significativamente reduzidas (p < 0,001).
8	*Nutritional status and associations with falls, balance, mobility and functionality during hospital admission* ^(23)^	2011 Austrália	Estudo observacional de coorte longitudinal. Foram incluídas as internações na Unidade de Avaliação e Reabilitação Geriátrica durante um período de seis meses.	Explorar as associações entre estado nutricional, quedas e fatores de risco selecionados entre idosos hospitalizados.	Os pacientes avaliados como desnutridos eram mais velhos (p<0,001) e mais propensos a ter uma pontuação pior no teste “*Up and Go*”, cronometrado tanto na admissão (p<0,05) quanto na alta (p<0,009). A desnutrição esteve associada à mobilidade reduzida (p<0,05).
9	*Ten Ways to Improve the Care of Elderly Patients in the Hospital* ^(24)^	2011 EUA	Teórico-conceitual	Não especificado	As formas de melhorar o atendimento ao paciente idoso hospitalizado incluem: triagem de síndromes geriátricas como delirium, avaliação do estado funcional e manutenção da mobilidade e implementação de intervenções que comprovadamente previnem delirium, quedas acidentais e declínio funcional agudo no hospital.
10	*A randomized controlled trial to prevent hospital readmissions and loss of functional ability in high risk older adults: a study protocol* ^(25)^	2011 Austrália	Ensaio controlado randomizado. 328 participantes (82/grupo)	Avaliar as estratégias inovadoras de cuidados de transição para reduzir readmissões não planeadas e melhorar o estado funcional, a independência e o bem-estar psicossocial de idosos comunitários em risco de readmissão.	Programas de exercícios individualizados projetados para melhorar a força, estabilidade, coordenação, resistência, mobilidade e autoconfiança em relação às atividades da vida diária. A prescrição de exercícios será desenvolvida usando uma abordagem de equipe envolvendo o paciente, cuidadores, médicos e enfermeiras da enfermaria.
11	*Predicting Habitual Physical Activity Using Coping Strategies in Older Fallers Engaged in Falls-Prevention Exercise* ^(26)^	2011 Inglaterra	Estudo observacional. 98 homens e mulheres	Examinar a atividade física em idosos que caem, aplicando uma teoria de adaptação, para determinar preditores de atividade física habitual.	Duas estratégias de enfrentamento, seleção baseada em perdas e otimização, explicaram melhor a mudança na atividade física entre o início e o acompanhamento.
12	*Improving physical activity in older adults receiving in patient rehabilitation: a phase II feasibility study* ^(27)^	2012 Austrália	Ensaio clínico randomizado (ECR) cego, único, com análise de intenção de tratar 47 participantes randomizados para um grupo controle (25) ou grupo de intervenção (22)	Avaliar a viabilidade de um ECR de aumento da atividade física em idosos em reabilitação.	O ECR proposto sobre aumento da atividade física em idosos em reabilitação era viável.
13	*Vestibular and Motor Contributions to Mobility: Limitations of Seniors Awaiting Discharge from Hospital Care* ^(28)^	2012 Canadá	Desenhos experimentais e de correlação	O objetivo principal deste artigo foi avaliar a capacidade de idosos hospitalizados de usar informações vestibulares para controle do equilíbrio. O objetivo secundário foi examinar a influência da função vestibular e da força muscular dos membros inferiores na mobilidade.	Idosos que aguardavam alta hospitalar apresentavam comprometimento do controle vestibular do equilíbrio, sistematicamente associado à dificuldade de mobilidade. A avaliação da função vestibular antes da alta hospitalar pode melhorar o planejamento da alta no que diz respeito ao manejo de deficiências que ameaçam o equilíbrio e a mobilidade segura.
14	*Measure, Promote and Reward Mobility to Prevent Falls in Older Patients* ^(29)^	2012	Teórico-conceitual	Não especificado	Deverá ser incentivada a aposta na manutenção e melhoria da mobilidade quando um idoso fica gravemente doente e é particularmente vulnerável à perda permanente da capacidade funcional durante uma hospitalização. Mais importante ainda, encorajar o treino rotineiro de força e equilíbrio em idosos residentes na comunidade deve ser uma prioridade.
15	*What is the involvement of the nursing team in maintaining and promoting the mobility of the elderly in the hospital? A grounded theory study* ^(30)^	2013 Inglaterra	Entrevistas semiestruturadas com 39 profissionais de reabilitação e 61 horas de observação não participante compuseram o conjunto de dados	Apresentar uma teoria fundamentada nos dados do envolvimento da equipe de enfermagem no processo de manutenção e promoção da mobilidade de idosos hospitalizados.	O envolvimento da equipe de enfermagem na manutenção e reabilitação da mobilidade dos pacientes foi explicado pela categoria central “cuidados para manter a segurança”. Identificou como a equipe de enfermagem se concentrou principalmente na prevenção de problemas do paciente em vez de focar nos objetivos de reabilitação. Uma série de fatores contextuais no local de trabalho fez com que a equipe de enfermagem tivesse dificuldade em se envolver em atividades de apoio à manutenção da mobilidade e à reabilitação.
16	*Activity restriction vs self-direction: hospitalised older adults’ response to fear of falling* ^(31)^	2014 EUA	Abordagem quantitativa e qualitativa combinada com extração de prontuários. 41 idosos	Descrever o medo de cair em idosos hospitalizados e sua relação com as características e função física do paciente e explorar a visão do paciente sobre os fatores associados.	Os participantes descreveram os seguintes fatores, organizados por enquadramento socioecológico, a serem considerados no desenvolvimento de alternativas à restrição de atividade: intrapessoais, interpessoais, ambientais e políticos.
17	*Implementing a Comprehensive Functional Model of Care in Hospitalized Older Adults* ^(32)^	2014 EUA	Amostra de conveniência. 866 idosos	Desenvolver um modelo abrangente de cuidados para promover a função física em idosos hospitalizados.	A implementação de um modelo funcional abrangente de cuidados para idosos hospitalizados teve um impacto positivo no tempo de internação, na taxa de readmissão em 30 dias e na taxa de quedas. A economia de custos estimada associada à redução do tempo de permanência pós-intervenção em 3 meses foi de aproximadamente US$ 200,00.
18	Diagnósticos e prescrições de enfermagem para idosos em situação hospitalar^(33)^	2015 Brasil	Pesquisa qualitativa submetida à análise estatística descritiva. 50 idosos	Identificar os diagnósticos de enfermagem descritos pela *North American Nursing Diagnosis Association* mais frequentes entre idosos em situação hospitalar e propor prescrições correlatas para idosos em situação hospitalar.	Dos idosos pesquisados, 36 (72%) apresentaram o diagnóstico Risco de quedas, manifestado pela necessidade de auxílio para deambulação e distúrbio de marcha. Pesquisa realizada por enfermeiras de Minas Gerais avaliou os fatores de risco apresentados para o diagnóstico de enfermagem Risco para quedas, observando o predomínio dos fatores intrínsecos sobre os extrínsecos. Os fatores intrínsecos de maior frequência foram idade acima de 65 anos (84%), uso de medicações (28%), dificuldade de marcha (22%) e história de quedas (22%).
19	*Effectiveness of an individualized fall prevention program in a geriatric rehabilitation hospital setting: a cluster randomized study* ^(34)^	2015 Israel	Ensaio controlado por *cluster* de dois estágios realizado em cinco enfermarias de reabilitação geriátrica. 752 pacientes	Investigar o efeito de um programa individualizado de prevenção de quedas em um hospital de reabilitação geriátrica.	Embora as quedas possam ocasionalmente ter uma explicação simples, são geralmente o resultado de interações sinérgicas entre fragilidades, doenças médicas de longa duração, doenças médicas agudas, medicamentos, o comportamento da própria pessoa e riscos ambientais.
20	*Moving forward in fall prevention: an intervention to improve balance among patients in a quasi-experimental study of hospitalized patients* ^(35)^	2015 Itália	Estudo prospectivo quase-experimental. 28 pacientes	Investigar se três programas de reabilitação diferentes, como exercícios em grupo, estabilidade central individual ou intervenção de treinamento de equilíbrio com uma plataforma estabilométrica, foram eficazes na melhoria da capacidade de equilíbrio em pacientes idosos hospitalizados e avaliar se houve diferenças em termos de eficácia entre esses três programas diferentes.	A participação em um programa de exercícios pode melhorar o equilíbrio e a mobilidade funcional, o que pode contribuir para a redução das quedas de pacientes idosos hospitalizados e dos subsequentes custos relacionados às quedas.
21	Instrumento de avaliação de quedas em idosos hospitalizados (IAQI Hospitalar): enfermeiro analisando vulnerabilidade e mobilidade^(36)^	2016 Brasil	Estudo exploratório e descritivo, com abordagem qualitativa	Elaborar um instrumento para avaliação da vulnerabilidade às quedas em idosos hospitalizados.	O IAQI auxilia na determinação do perfil individual e vulnerabilidade do idoso, para que sejam programadas ações de prevenção das quedas.
22	*Comparison of posthospitalization function and community mobility in hospital mobility program and usual care patients: a randomized clinical trial* ^(37)^	2016 EUA	Ensaio clínico randomizado simples-cego. 100 pacientes	Examinar o efeito de um programa de mobilidade (PM) intra-hospitalar na função pós-hospitalização e na mobilidade comunitária.	A intervenção PM permitiu que os pacientes mantivessem a mobilidade comunitária pré-hospitalização, enquanto aqueles no grupo de cuidado usual experimentaram declínios clinicamente significativos. A menor mobilidade no espaço de vida está associada ao aumento do risco de morte, admissão em lares de idosos e declínio funcional, sugerindo que declínios como os observados no grupo de cuidado usual seriam de grande importância clínica.
23	*The effects of a high-intensity functional exercise group on clinical outcomes in hospitalised older adults: an assessor-blinded, randomised-* *controlled trial* ^(38)^	2017 Austrália	Ensaio mono-cego, randomizado e controlado. 468 pacientes	Investigar um grupo de exercícios funcionais de alta intensidade (HIFE, sigla em inglês) em idosos hospitalizados.	Os resultados sugerem que um programa de grupo HIFE combinado com fisioterapia individual pode melhorar a mobilidade de forma semelhante à fisioterapia individual isolada em idosos hospitalizados. Fornecer fisioterapia em grupo resultou em maior eficiência do terapeuta. Um grupo de exercícios de alta intensidade com fisioterapia individual pode ser um método eficaz e eficiente para prestar cuidados a pacientes idosos internados.
24	*The Case for Mobility Assessment in Hospitalized Older Adults: American Geriatrics Society White Paper Executive Summary* ^(4)^	2018 EUA	*White paper* que apoia um maior foco na mobilidade como resultado para idosos hospitalizados	Avaliar e prevenir a perda de mobilidade em idosos no hospital e resumir as recomendações desse *white paper*.	1) Promover a avaliação da mobilidade em cuidados agudos; 2) Defender mais financiamento para pesquisa; 3) Desenvolver consenso sobre métodos padrão para avaliar a mobilidade; 4) Minimizar o peso da medição da mobilidade; 5) Avaliar a viabilidade de uma medida de qualidade de mobilidade; 6) Reestruturar o atual foco regulatório nas quedas nos cuidados intensivos para um foco na mobilidade segura; 7) Desenvolver recursos para prestadores de cuidados intensivos.
25	*Muscle strength is longitudinally associated with mobility in the elderly after acute hospitalization: the Hospital-ADL study* ^(39)^	2019 Países Baixos	Estudo de coorte multicêntrico, prospectivo e observacional. 391 idosos	Determinar (i) o curso da mobilidade de idosos hospitalizados em situação aguda e (ii) a associação entre a força muscular e o curso da mobilidade ao longo do tempo controlado por fatores de influência.	A força muscular está longitudinalmente associada à mobilidade. São necessárias intervenções para melhorar a mobilidade, incluindo a força muscular, em idosos hospitalizados em estado agudo.
26	*An augmented prescribed exercise program (APEP) to improve mobility in older acute medical patients - a randomized controlled pilot and feasibility study* ^(40)^	2019 Alemanha	Piloto controlado e estudo de viabilidade unicêntrico, prospectivo, de grupo paralelo, cego, randomizado (1:1). 35 participantes	Avaliar a viabilidade de um programa aumentado de exercícios prescritos (APEP, sigla em inglês) em pacientes médicos agudos mais velhos e medir os efeitos potenciais do APEP na capacidade de mobilidade, a fim de avaliar a viabilidade de um grande estudo em grande escala.	Este pequeno ECR de viabilidade indica que uma intervenção APEP pode ser segura e viável em pacientes clínicos agudos mais velhos. A APEP pode possivelmente induzir efeitos pequenos a moderados na mobilidade, mas a relevância clínica destes efeitos pode ser limitada.
27	*Optimizing Function and Physical Activity in Hospitalized Older Adults to Prevent Functional Decline and Falls* ^(41)^	2019 EUA	Teórico-conceitual	Não especificado.	Aumentar a atividade física dos pacientes e diminuir as quedas é extremamente importante para otimizar os resultados dos pacientes e diminuir o tempo de internação hospitalar. Não existe uma abordagem única que garanta efetivamente o tempo ideal gasto em atividade física ou que não ocorra uma queda. São necessárias abordagens multifatoriais que se concentrem nos riscos e desafios individuais de cada indivíduo.
28	*Predictors of physical activity in older adults at the start of an emergency hospital stay: a prospective cohort study* ^(42)^	2020 Reino Unido	Análise secundária de estudo prospectivo de coorte de medidas repetidas. 62 participantes	Investigar preditores de atividade intra-hospitalar durante um período de 24 horas nas primeiras 48 horas de internação hospitalar em idosos.	A atividade física, particularmente na fase aguda da hospitalização, é muito baixa nos idosos. A associação entre a gravidade da doença e a atividade física pode ser explicada pelo facto de os sintomas da doença aguda serem barreiras à atividade.
29	*Promoting mobility among hospitalized elderly: an exploratory study on the perceptions of patients, caregivers and nurses* ^(43)^	2020 Singapura	Estudo qualitativo descritivo com abordagem amostral proposital e por conveniência. 14 pacientes, seis cuidadores e dez enfermeiros	Explorar as percepções dos pacientes e dos seus cuidadores, bem como dos enfermeiros sobre a promoção da mobilidade entre pacientes idosos adultos hospitalizados.	O reconhecimento da importância da mobilidade, bem como dos efeitos prejudiciais do repouso prolongado no leito durante a hospitalização, é um primeiro passo essencial para o desenvolvimento de intervenções bem-sucedidas para promover a mobilidade no contexto asiático. É necessário fazer melhorias para ajudar a superar as várias barreiras e desafios na mobilização dos pacientes idosos nos ambientes de cuidados intensivos. Os enfermeiros e outros membros da equipe de cuidados podem ajudar a aumentar a confiança dos pacientes e dos prestadores de cuidados familiares (na prestação de assistência durante a mobilidade), através de modelos e fornecimento de auxiliares de marcha, bem como de educação baseada no risco.
30	*Factors associated with walking in hospitalized elderly* ^(44)^	2020 Irlanda	Estudo de coorte. 154 participantes	Identificar as características do paciente na admissão e os eventos diários durante a internação que poderiam influenciar a atividade de caminhada dos pacientes idosos durante a internação.	A contagem diária de passos flutuou, sugerindo uma margem considerável para intervenção. Os dispositivos ou tratamentos que dificultam a marcha devem ser revistos diariamente e a atividade de caminhada deve tornar-se uma prioridade clínica. O desempenho físico na admissão pode identificar pacientes vulneráveis.
31	*Assisted Walking Program on Walking Ability in In-Hospital Geriatric Patients: A Randomized Trial* ^(45)^	2021 Itália	ECR. 387 pacientes	Avaliar se um programa individualizado de caminhada assistida para pacientes idosos hospitalizados poderia melhorar a capacidade de locomoção em comparação com cuidados geriátricos e reabilitação habituais.	As características basais foram semelhantes entre os grupos intervenção e controle. O grupo de intervenção, em relação ao grupo controle, melhorou significativamente a capacidade de caminhar na alta (P < 0,001). Não houve diferenças estatisticamente significativas entre os grupos em termos de eventos adversos intra-hospitalares. Nenhum efeito adverso foi detectado.
32	*Reimagining Injurious Falls and Safe Mobility* ^(2)^	2021 EUA	Teórico-conceitual	Propor uma nova abordagem para reduzir quedas com lesões em idosos baseada em protocolos baseados em evidências conhecidos por impactar positivamente a saúde dos idosos.	ERA (sigla em inglês) - Integração de registros eletrônicos de saúde, Fatores de risco importantes, Avaliação e planos de cuidados - permite que os enfermeiros usem uma ferramenta validada de avaliação de risco de queda para reformular os fatores de risco de queda como parte do plano de cuidados abrangente e mapear fatores de risco modificáveis para intervenções que abordem as causas subjacentes às quedas e promover uma mobilidade mais segura. A abordagem ERA pode ajudar os enfermeiros a utilizar o seu tempo de forma mais eficaz, concentrando-se em ações específicas que melhoram os resultados dos pacientes, trabalhando em coordenação com uma equipe interprofissional e interprofissional de cuidados contínuos.
33	*Effects of General Physical Activity Promoting Interventions on Functional Outcomes in Patients Hospitalized over 48 Hours: A Systematic Review and Meta-Analysis of Randomized Controlled Trials* ^(46)^	2021 Países Baixos	Estudo de revisão sistemática: cinco bases de dados eletrônicas foram pesquisadas em busca de ECR. Para resultados relatados em dois estudos ou mais, foi realizada meta-análise para testar diferenças entre grupos	Avaliar o efeito da atividade física geral, promovendo intervenções nos resultados funcionais e hospitalares em pacientes hospitalizados por mais de 48 horas.	No geral, não foram encontradas evidências conclusivas sobre o efeito das intervenções de promoção da atividade física geral nos resultados funcionais.
34	*Pilot testing of nurse led multimodal intervention for falls prevention* ^(47)^	2022 EUA	Estudo piloto prée pós-teste em grupo único. 70 pacientes	Examinar o efeito de uma intervenção multimodal liderada por enfermeiros nos níveis de consciência do risco de queda, autoeficácia e envolvimento na prevenção de quedas de adultos hospitalizados.	Houve diferenças significativas [pré-teste (M= 19,02, DP=1,152) e pós-teste (M= 21,71, DP=0,527); t (17,355) = 58, p.001] sobre o nível de consciência do risco de queda na prevenção de quedas. Não houve resultados significativos para autoeficácia e engajamento em quedas. Os resultados do estudo sugeriram que, quanto maior a autoeficácia em quedas, maior o envolvimento. Pesquisas futuras são necessárias para examinar a autoeficácia e o envolvimento na prevenção de quedas em coortes mais amplas e diversificadas de idosos hospitalizados.
35	*Effect of a Ward-Based Program on Hospital-Associated Complications and Length of Stay for Older Inpatients The Cluster Randomized CHERISH Trial* ^(48)^	2022 Austrália	Ensaio randomizado de *cluster* pragmático. 539 pacientes	Implementar e avaliar um programa de melhoria baseado em enfermarias (“*Eat Walk Engage*”) para fornecer de forma mais consistente princípios de atendimento amigáveis aos idosos para idosos em situações agudas em enfermarias de pacientes.	*Eat Walk Engage* não reduziu o desfecho primário composto de qualquer HAC-OP (sigla em inglês, complicações associadas ao hospital em idosos), que afetou quase metade dos pacientes idosos internados, embora tenha havido uma redução significativa no *delirium*.

Nenhum dos estudos apresentou o conceito claro de mobilidade segura, entretanto foi evidenciada a preocupação de que a mobilidade deve ser promovida de modo a garantir a segurança do paciente, conforto, qualidade de vida e prevenir a alta dependência^(43)^. Apesar de o conceito não ser preciso, foram identificados elementos constitutivos do conceito, que estão relacionados ao paciente, à instituição e à natureza das intervenções, conforme Figura 2.

**Figura 2:** Elementos constitutivos do conceito de mobilidade segura em pessoas idosas hospitalizadas relacionados ao paciente, à instituição e à natureza das intervenções, Rio de Janeiro, Rio de Janeiro, Brasil, 2023

## DISCUSSÃO

A extensão das evidências disponíveis mapeadas nesta revisão de escopo mostrou que os fatores intrínsecos relacionados ao paciente são os citados com maior frequência no que tange à promoção da mobilidade. Os fatores comportamentais^(2,16,19,21,27,29,31,33-36,39-40,43,46-47)^ são os mais prevalentes, sendo mencionados em cerca de 43% dos textos (n=15). Entre eles, comportamento sedentário^(31)^ e inatividade^(46)^, isolamento social e solidão^(2)^, desmotivação^(46)^, sintomas depressivos^(31,39,41)^, medo de cair^(21,31,37,39,41,44)^ e preocupações com as lesões que as quedas podem acarretar^(44)^, além de crenças e perspectivas sobre a mobilidade^(37)^, como associar a ideia de estar hospitalizado à permanência no leito para recuperação da saúde ou de que é uma regra da instituição que os pacientes devam permanecer em seus quartos, despontam como fatores que limitam a confiança da pessoa idosa, tornando-se obstáculos para a promoção da mobilidade.

Nesse sentido, o alcance da mobilidade segura pressupõe avaliar os fatores comportamentais, propondo intervenções multifacetadas, com estímulo ao convívio social^(29,33,47)^, como refeições em grupo ou atividades durante o dia, em conjunto e fora do quarto, e orientações verbais e escritas ao paciente^(24,30-32,43)^ tendem a motivá-lo^(41,45)^ e encorajá-lo^(30-31,45)^. Como estratégia de enfrentamento, podem ser traçadas metas progressivas^(41)^, incentivando-o inicialmente a desempenhar suas atividades básicas da vida diária de forma independente^(25)^. Ademais, é importante o envolvimento multiprofissional, uma vez que pacientes repetidamente advertidos por saírem do leito evocam comportamentos agressivos, além de impedir iniciativas da pessoa idosa^(22)^.

Outro conjunto de fatores associados à mobilidade segura são condições e doenças agudas e crônicas^(16,19,22,28,34,36,41,43,46-47)^. Apesar de um estudo revelar que não houve influência das comorbidades na relação entre força muscular e mobilidade^(39)^, os demais autores apresentam que condições (fragilidade)^(34)^, patologias (incontinência urinária, deficiência visual^(28,37,41-42,44,46)^, anemia^(41)^, *delirium* e demência^(22,32,36,39,41)^, entre outras), bem como os sinais e sintomas delas advindos^(16,19,28,34,36,41-42,46-47)^ (falta de ar^(46)^, instabilidade respiratória e cardiovascular^(42)^, fadiga^(37,39,42,44)^, fraqueza^(37,44)^ e dor^(37,40-42,44,46)^), levam à condição de incapacidade ou aumentam o risco de queda. Dessa forma, para mobilidade segura, é necessário reconhecer o impacto das doenças e seu manejo adequado, ponderando, entretanto, dois outros fatores relevantes: dispositivos para tratamento^(5,31,37,41-42,44)^ e medicamentos e polifarmácia^(16,34,42)^.

Cateter endovenoso e equipo^(5,30,37,41,43)^, cateter vesical de demora^(5,31,37,41)^, oxigênio na rede de gases^(41,44)^, monitorização^(41)^ e drenos^(46)^ são dispositivos de tratamento frequentemente utilizados em unidades hospitalares, com vistas à recuperação da saúde, que, entretanto, prejudicam a mobilidade. Dessa forma, propõe-se a remoção precoce de cateteres endovenosos, a interrupção momentânea de infusões lentas ou a utilização de suporte de soro com rodinhas, para que esse possa ser transportado com segurança^(44)^, e a oferta de oxigênio suplementar através de pequenos cilindros portáteis^(44)^.

Quanto à terapia medicamentosa^(16,34,41)^, essa deve ser avaliada, atentando-se particularmente à polifarmácia^(41)^ e à prescrição de sedativos, psicotrópicos, diuréticos e hipotensores^(24)^, fármacos cujos efeitos tornam as pessoas idosas mais vulneráveis à queda^(2,41)^. Quanto maior o número de medicamentos em uso, menor o nível de autoeficácia e engajamento na prevenção de quedas^(47)^. Destarte, deve-se considerar a complexidade do manejo da prevenção de quedas e mobilidade segura de pessoas idosas hospitalizadas, que somam às condições de senescência e senilidade, e que, se não propostas adequadamente, podem vir a agravar o estado do paciente.

Força muscular^(23,36,38)^, qualidade da marcha^(36)^ e equilíbrio^(27-28,36)^ também são pré-requisitos essenciais para a mobilidade segura. O comprometimento da força muscular e o equilíbrio culminam em instabilidade postural e, portanto, em predisposição para quedas^(36)^. Dessa forma, propõe-se a realização de exercícios^(23,39-40)^ que melhorem a força, a estabilidade do core, coordenação, resistência^(38)^ e mecânica corporal, desenvolvendo a confiança^(25)^, tais como programas de exercícios estruturados^(24)^ individuais ou em grupo, de equilíbrio^(26)^ e treino em plataforma estabilométrica^(27)^, além de exercícios de sentar e levantar, progredindo para mudança de peso, em um lugar fixo e depois deambulando^(37)^, fortalecimento com peso^(26)^, plano de caminhada^(26)^ e exercícios articulares^(33)^.

Para a realização dos exercícios, deve-se considerar duas questões. A primeira é o suporte nutricional. Enquanto um estudo revelou que o Índice de Massa Corporal e o estado nutricional não exerceram influência na relação entre a força muscular e a mobilidade^(39)^, outros, por outro lado, revelam que a obesidade e a desnutrição^(22,36,40-42)^ estão associadas à perda muscular, a uma pior função física e consequente risco de queda, e, por isso, a mobilização deve ser escrupulosa^(42)^. Nesse sentido, particularmente em exercícios com treinamento de resistência e força, o suporte nutricional é essencial, a fim de que não haja perda ponderal^(22)^.

A segunda questão é que os exercícios sejam planejados ponderando a não maleficência. A promoção de atividade física é uma intervenção simples e não invasiva, com potencial de melhorar a mobilidade^(26)^, entretanto, para que seja segura, deve ser dosada para que não provoque sintomas como dispneia, fraqueza e fadiga^(31)^. A atividade física segura previne a perda da função e da reserva fisiológica por imobilidade e acelera a restauração das funções perdidas devido a doenças agudas^(16,46)^, cooperando, inclusive, para a mobilidade comunitária pós-alta^(18)^. Recomenda-se, inclusive, que a mobilização seja iniciada precocemente^(21,24,32,45,47)^, desde o momento da hospitalização, a partir de uma avaliação inicial de risco^(30)^, para que o nível de funcionalidade pré-internação seja mantido^(16)^, já intencionando o planejamento de alta^(28,40)^.

Ocorrem, contudo, divergências na literatura quanto à designação do profissional responsável pela promoção da mobilidade segura, particularmente em pacientes mais dependentes e que exigem o amparo de um profissional^(37)^. É consenso geral que a abordagem interprofissional e de cuidados contínuos é salutar tanto para o sistema quanto para o paciente^(2,16,22,29,42,48)^, entretanto os textos destacam o protagonismo de fisioterapeutas e enfermeiros.

Fisioterapeutas promovem sessões com avaliação e tratamentos que maximizam a mobilidade e independência a partir da prescrição e entrega de exercícios, concorrendo para a confiança da pessoa idosa conforme evolução e acompanhamento^(21,38,40)^. Entende-se, entretanto, a partir das leituras, que são intervenções pontuais.

Enfermeiros com maior proximidade com o paciente são fundamentais para assistir e supervisionar diretamente os pacientes na promoção da mobilidade (mover-se, sair do leito, deambular)^(30,33,44)^. Entretanto, algumas questões apontadas pelos enfermeiros precisam ser destacadas: 1) Falta pessoal e enfermagem sobrecarregada^(4,42)^ - há a necessidade de maior presença da fisioterapia^(37)^; 2) Os enfermeiros não se sentem capacitados para fornecer assistência física e avaliá-la adequadamente^(42)^ (Essa é uma das razões pela qual a deambulação não é encorajada rotineiramente, provocando, por sua vez, uma excessiva dependência da fisioterapia^(4)^, bem como são prestadas orientações equivocadas, como repouso no leito ordenado^(44)^, mesmo para pacientes que não precisam de auxílio. Ademais, muitos enfermeiros não compreendem a validade, confiabilidade e utilidade de medidas de avaliação da mobilidade^(4)^, assim como não há processos padronizados e validados para encorajar a mobilidade segura da pessoa idosa durante a internação^(4)^); 3) Alguns enfermeiros não identificam a promoção da mobilidade como sua responsabilidade, não colaborando com fisioterapeutas e adiando orientações^(4)^.

Por fim, compreende-se que a enfermagem auxilia no processo de cuidados físicos e pessoais, amparando na movimentação, transferência e atividades básicas, que necessitam de supervisão^(30)^. A avaliação de enfermagem é importante na identificação de síndromes geriátricas^(24)^, assim como na avaliação e reconhecimento de fatores de risco de queda, que devem ser contemplados em um plano de cuidados, além de riscos relacionados ao ambiente^(2)^. A fisioterapia, por sua vez, é especialista em movimento, manuseio e reabilitação, cabendo a ela tais funções^(30)^.

Ainda quanto aos desafios para a promoção da mobilidade segura, insta salientar a falta de investimentos financeiros e de equipamentos, como auxiliares de marcha^(4,31,37,44,46)^, que possibilitem maior liberdade e segurança^(36)^. Uma outra possibilidade são os recursos de gerontecnologia, como cintos de marcha^(19)^, protetores de quadril^(17-18)^, andador inteligente^(17-18)^, alarmes de queda^(17-18)^, tapete antiderrapante^(18)^ e sensores de movimento^(6)^. É interessante que a equipe de enfermagem esteja à frente da seleção e teste dos equipamentos, integrando tecnologias novas à infraestrutura existente, de modo que os efeitos adversos relacionados à mobilidade sejam eliminados ou mitigados, o que favorece o tratamento, a reabilitação e a segurança do paciente^(17)^.

Nesse sentido, é importante que o ambiente seja melhorado^(17,34,43)^, como altura da cama e dos móveis adequada^(24,27,31)^; que a estrutura da enfermaria possibilite visualizar vários pacientes ao mesmo tempo^(41)^, com corredores abertos que facilitem a deambulação^(41)^, tapetes de alto impacto e pisos acolchoados que minimizem o risco de lesão^(24,29,41)^; que haja iluminação adequada de corredores e quartos^(22,32)^; e que o hospital seja sinalizado e sejam disponibilizados mapas, de modo que o paciente possa se orientar ao deambular pela instituição^(36,48)^.

Por fim, deve-se oferecer vestimentas^(19)^ e calçados adequados^(22,32)^, atentar-se à qualidade do sono^(42,44)^, ao tempo de internação^(20)^, à idade, pois o avançar da idade contribui para um maior risco de queda^(33)^, e integrar a família aos cuidados da pessoa idosa, tornando-os facilitadores e defensores^(31)^ da mobilidade segura, apoiando e reduzindo sentimentos negativos^(2,22)^.

Destarte, o mapeamento das evidências mostrou que a mobilidade segura está relacionada à natureza das intervenções e a fatores de risco não modificáveis e modificáveis relacionados ao paciente, sendo que os fatores de risco modificáveis são passíveis de intervenção. Os aspectos relacionados à instituição abarcam a capacitação e qualificação de profissionais, dimensionamento de pessoal adequado, investimento em equipamentos, tecnologias e reformas estruturais, além de oferecer vestimentas e calçados adequados e convergentes à ideia.

Observou-se, durante o mapeamento dos estudos, a valorização da promoção da mobilidade da pessoa idosa, assim como da mobilização precoce, contudo poucos estudos se preocuparam em estudar como realizá-la com segurança. Nesse sentido, tal lacuna indica a necessidade de sejam realizados mais estudos que destaquem a segurança do paciente.

### Limitações do estudo

As limitações deste estudo envolvem a limitação das abordagens dos estudos sobre as dimensões da vida da pessoa idosa que podem influenciar na mobilidade segura e no risco de acidentes por queda, tais como aspectos cognitivos, de estado mental e emocional, além de não aprofundar na terapia medicamentosa. Ademais, as limitações podem estar relacionadas à busca em um número singelo de fontes de literatura cinzenta.

### Contribuições para a área da saúde

A contribuição da revisão para o campo da saúde aponta mudanças na percepção da prevenção de quedas em pessoas idosas hospitalizadas ao longo dos últimos anos. Estudo realizado em 2007 defende que, até que dados sobre estratégias bem-sucedidas estivessem disponíveis, a minimização da mobilidade poderia permanecer como a solução padrão para a prevenção de quedas^(19)^, enquanto estudo de 2021 compreende que a preservação da mobilidade e independência exige alguns riscos de quedas^(2)^. As intervenções testadas que obtiveram maior êxito estão relacionadas à promoção de exercícios de fortalecimento muscular e equilíbrio^(27-29,32,35,38-39,41,45)^.

## CONSIDERAÇÕES FINAIS

As evidências acerca dos elementos constitutivos da mobilidade segura sustentam que esses estão relacionados ao paciente (fatores comportamentais, condições, doenças agudas e crônicas, sinais e sintomas, estado nutricional, idade, equilíbrio, força muscular e qualidade de marcha e padrão de sono), à instituição (riscos ambientais, dispositivos para tratamento, orientações equivocadas, medicamentos e polifarmácia, recursos, vestimentas e calçados) e à natureza das intervenções (relacionadas à família, ao paciente e à instituição), admitindo-se que maiores possibilidades de intercessão estão relacionadas à última e envolvem múltiplas dimensões. Nesse sentido, a mobilidade segura é uma expressão da aptidão das unidades hospitalares para garantir cuidado e proteção de acidentes por queda a pessoas idosas. A presente revisão evidenciou, entretanto, que os recursos de preparação do ambiente e dos profissionais de saúde para lidar com demandas específicas da população idosa são hipossuficientes. Por fim, fica sensibilizado sugerir a realização de uma análise de conceito do termo “mobilidade segura”.
